# mHealth use for non-communicable diseases care in primary health: patients’ perspective from rural settings and refugee camps

**DOI:** 10.1093/pubmed/fdy172

**Published:** 2018-10-11

**Authors:** Shadi Saleh, Angie Farah, Nour El Arnaout, Hani Dimassi, Christo El Morr, Carles Muntaner, Walid Ammar, Randa Hamadeh, Mohamad Alameddine

**Affiliations:** 1 Department of Health Management and Policy, Faculty of Health Sciences, American University of Beirut, Riad El Solh, Beirut, Lebanon; 2 Global Health Institute, American University of Beirut, Riad El Solh, Beirut, Lebanon; 3 Department of Pharmaceutical Sciences, School of Pharmacy, Lebanese American University, Beirut, Lebanon; 4 School of Health Policy & Management, Faculty of Health, School of Health Policy and Management, York University, 4700 Keele St., Toronto ON, Canada; 5 Social & Behavioural Health Sciences Division, Dalla Lana School of Public Health, University of Toronto, Toronto, Ontario, Canada; 6 Ministry of Public Health, Ministry of Public Health, Beirut, Lebanon; 7 Mohammed Bin Rashid University of Medicine and Health Sciences, Dubai Healthcare City, Dubai, United Arab Emirates

**Keywords:** e-health, refugees

## Abstract

**Background:**

Non-communicable diseases (NCDs) account for 85% of deaths in Lebanon and contribute to remarkable morbidity and mortality among refugees and underserved populations. This study assesses the perspectives of individuals with hypertension and/or diabetes in rural areas and Palestinian refugee camps towards a population based mHealth intervention called ‘eSahha’.

**Methods:**

The study employs a mixed-methods design to evaluate the effectiveness of SMSs on self-reported perceptions of lifestyle modifications. Quantitative data was collected through phone surveys, and qualitative data through focus group discussions. Descriptive statistics and bivariate analysis were performed.

**Results:**

About 93.9% (*n* = 1000) of respondents perceived the SMSs as useful and easy to read and understand. About 76.9% reported compliance with SMSs through daily behavioral modifications. Women (*P* = 0.007), people aged ≥76 years (*P* < 0.001), unemployed individuals (*P* < 0.001), individuals who only read and write (*P* < 0.001) or those who are illiterate (*P* < 0.001) were significantly more likely to receive and not read the SMSs. Behavior change across settings was statistically significant (*P* < 0.001).

**Conclusion:**

While SMS-based interventions targeting individuals with hypertension and/or diabetes were generally satisfactory among those living in rural areas and Palestinian refugee camps in Lebanon, a more tailored approach for older, illiterate and unemployed individuals is needed.

## RESUMEN

**Contexto:**

En el Líbano, las enfermedades no transmisibles (ENT) representan el 85 % de las muertes y agravan los altos niveles de morbilidad y mortalidad entre los refugiados y los grupos de población subatendidos. Este estudio evalúa las opiniones de personas con hipertensión y/o diabetes que viven en zonas rurales y en campos de refugiados palestinos con respecto a una intervención de salud móvil basada en la población denominada ‘eSahha’.

**Métodos:**

El estudio emplea un diseño de métodos mixtos para evaluar la eficacia de los SMS sobre los cambios en el estilo de vida, según los perciben los propios interesados. Se obtuvieron datos cuantitativos mediante encuestas telefónicas y datos cualitativos a través de debates en grupos de sondeo. Se realizaron estadísticas descriptivas y un análisis bivariante.

**Resultados:**

Alrededor del 93,9 % (*n* = 1000) de los encuestados opinaron que los SMS son útiles y fáciles de leer y comprender. Un 76,9 % manifestó cumplir con los SMS mediante modificaciones en su comportamiento cotidiano. Las mujeres (*P* = 0,007), las personas mayores de 76 años (*P* < 0,001), las personas desempleadas (*P* < 0,001), las personas que solo sabían leer y escribir (*P* < 0,001) o aquellas que eran analfabetas (*P* < 0,001) tenían muchas más probabilidades de recibir y no leer los SMS. Los cambios de comportamiento en los distintos entornos tuvieron una importancia estadística (*P* < 0,001).

**Conclusión:**

Aunque, en general, las intervenciones por SMS orientadas a personas con hipertensión y/o diabetes fueron satisfactorias entre quienes vivían en zonas rurales y en campos de refugiados palestinos del Líbano, se necesita un enfoque más ajustado para las personas mayores, analfabetas y desempleadas.

**Palabras clave:**

cibersalud, refugiados

## RÉSUMÉ

**Contexte:**

Les maladies non transmissibles sont à l’origine de 85 p. 100 des décès au Liban et contribuent à une morbidité et une mortalité graves chez les réfugiés et les populations mal desservies. Cette étude analyse le point de vue des personnes souffrant d’hypertension ou de diabète dans les régions rurales et dans les camps de réfugiés palestiniens en ce qui a trait à une intervention en santé mobile axée sur la population appelée « eSahha ».

**Méthodes:**

L’étude a recours à un modèle regroupant plusieurs méthodes pour évaluer l’efficacité des messages textes sur la façon dont les personnes disent percevoir la modification de leur mode de vie. Des données quantitatives ont été recueillies par des sondages téléphoniques, et des données qualitatives par des discussions thématiques de groupe. L’étude comprend la réalisation de statistiques descriptives et d’analyses bivariées.

**Résultats:**

Près de 93,9 p. 100 (*n* = 1000) des personnes interrogées perçoivent les messages textes comme utiles et faciles à lire et à comprendre. Environ 76,9 p. 100 ont déclaré se conformer aux messages textes en modifiant chaque jour leur comportement. Les femmes (*P* = 0,007), les personnes âgées ≥76 ans (*P* < 0,001), les personnes sans emploi (*P* < 0,001), les personnes qui ne savent que lire et écrire (*P* < 0,001) ou celles qui sont analphabètes (*P* < 0,001) sont beaucoup plus disposées à recevoir, mais à ne pas lire les messages textes. Une modification de comportement tous environnements confondus était statistiquement significative (*P* < 0,001).

**Conclusion:**

Les interventions par messages textes qui ciblent les personnes qui souffrent d’hypertension ou de diabète étaient en général satisfaisantes pour les personnes vivant dans les régions rurales et dans les camps de réfugiés palestiniens au Liban; toutefois, il faut une approche plus adaptée pour les personnes âgées, analphabètes et sans emploi.

**Mots clés:**

cybersanté, réfugiés

## Background

Non-communicable diseases (NCDs), particularly diabetes and hypertension, are among the most significant global health challenges, with the greatest burden found in developing countries, where 80% of all NCD-related deaths occur.^1–5^ In light of this, Primary Health Care (PHC) has received renewed attention, as the most effective strategy targeting NCDs.^1,2,6^ However, in many low- and middle-income countries (LMICs), access to PHC by disadvantaged populations remains limited.^2,5,7^ The situation is aggravated among populations affected by conflict especially in some Middle Eastern countries with high rates of diabetes and hypertension, especially among vulnerable populations and refugees.^5,8,9^

Mobile health (mHealth), a subset of elec/tronic health (eHealth), is defined as the use of mobile devices in healthcare delivery, mainly through short message service (SMS), voice calls, and smartphone applications, among others. Several studies underlined the effectiveness of mHealth tools in delivering public health interventions, with the ability to overcome the financial and geographic barriers facing hard-to-reach populations.^4,10–12^

There is evidence from various countries regarding the potential of mHealth in addressing NCDs through health education and self-management, improving prevention and treatment strategies, and providing appointment reminders to improve compliance and attendance in PHC.

Despite being a basic mHealth tool, SMSs are increasingly used in healthcare due to their feasibility, low-cost, ease of use, convenience, independence of internet access and minimal requirement of technological literacy.^10–14^ Evidence on patients’ experiences with SMSs were identified in several studies reflecting high acceptability of and satisfaction with the service as well as the usefulness of SMSs for behavior change among diabetic and hypertensive patients.^10,11,13–22^

In Lebanon, NCDs account for 85% of all deaths,^23^ and are the main causes of morbidity and mortality among Palestinian refugees.^9^ A primary challenge among underserved populations in rural areas and refugee settings is the high burden of NCDs coupled with limited access to healthcare.^5,8^ Frequently sub-optimally resourced PHC Centers (PHCCs) are the only facilities available in underprivileged and rural areas.^8^ The globalization of unhealthy behaviors and lifestyles,^24^ as well as certain social determinants of health such as education^25–28^ and income levels,^29,30^ further underscore the importance of researching NCDs in Lebanon.

Despite the inability of some studies to statistically demonstrate improved health outcomes upon employment of mHealth interventions,^31^ several other studies showed that SMS-based mHealth interventions led to better health outcomes in LMICs, especially in resource-poor settings.^10,12,32,33^ This is supported by an exponential growth witnessed in the number of mobile phone users in LMICs, including Lebanon where 86% of the population own a cell phone, with sending and receiving text messages being the most popular use (89%).^34^ This offers a promising ground for the use of mHealth for disease education and behavior change, which has driven the implementation of the eSahha project.

The eSahha project is a two-pronged mHealth interventional project targeting sixteen PHCCs in six different Lebanese governorates. The centers were randomly assigned into intervention and control groups, for a total of eight sites in each of the groups. Among each of intervention and the control groups, five PHCCs were located in rural areas, and three PHCCs were located in Palestinian refugee camps. All rural and refugee settings included were considered underserved areas with limited access to health knowledge and healthcare.

The implementation of the intervention was conducted in partnership with the Lebanese Ministry of Public Health (MOPH) and the United Nations Relief and Works Agency for Palestine Refugees in the Near East (UNRWA).

The community-based intervention aimed to enhance access among underserved rural and refugee populations to health services specific to hypertension and/or diabetes. Community screening for hypertension and diabetes was conducted among the high risk group—40 years and above—in the catchment areas of the intervention PHCCs. Individuals already diagnosed with or suspected of being diabetic, hypertensive or both were referred to the nearest intervention PHCC for NCD-specific clinical care and were targeted by SMS messages originating from a pre-existing mass messaging platform hosted by a Lebanese telecommunication company. The messages were sent to the mobile phones of targeted individuals suffering from hypertension and/or diabetes included in the intervention or, in cases where the latter didn’t own a mobile phone, messages were sent to the cell phones of their respective closest relatives (e.g. son, daughter, husband, etc.). A weekly educational SMS was sent every Monday afternoon for the intervention period of 1 year. SMSs covered different health themes providing health information on lifestyle, dietary habits, body weight, smoking, medications, and symptoms of hypertension and diabetes (Table 1). In parallel, individuals using the services of the control PHCCs, did not receive community screening and referral services, nor were they enrolled in the SMS intervention.

**Table 1:** Examples of the Content of Text Messages Sent to Patients

SMS theme	SMS example
Follow-up	‘For all diabetics, it is important to control your weight and lose any extra kilos for better control of your condition’ (diabetes)
	‘Follow up with your doctor and perform the needed laboratory and EKG tests periodically’ (hypertension)
Diet	‘Eating less fat controls blood lipids and helps in losing weight. Grill, steam or bake instead of frying or oil cooking’ (diabetes)
	‘Calcium found in dairy foods helps to regulate your blood pressure. Opt for low-fat milk, yogurt, and cheese’ (hypertension)
Stress	‘Stress exacerbates diabetes since it can raise blood glucose levels leading to eye and kidney disorders’ (diabetes)
Physical activity	‘To better control the blood pressure, stay physically active. The daily recommendation is 30 minutes in three 10-minute segments’ (hypertension)

SMS, Short Message Service; EKG, electrocardiogram.

For individuals who were already diagnosed and receiving necessary care, personalized SMSs were sent as reminders of their scheduled appointments, labs such as HbA1C levels, and/or their annual foot or eye exams, among others. Individuals in the control group did not receive SMSs and were thus receiving the usually practiced care in the control PHCCs. Based on the MOPH guidelines for prevention and management of hypertension and diabetes, the SMSs were developed by a family physician and fine-tuned based on results of pilot testing of the SMSs with community representatives. The messages, up to 70 characters in length, were initially formulated in English and then translated and sent using simplified Arabic terms to match the target population’s different levels of health literacy.

This study is part of a series of manuscripts evaluating the eSahha intervention.^35,36^ It aims to assess the views and attitudes of hypertensive and/or diabetic individuals enrolled in the eSahha intervention and residing in Lebanese rural areas and Palestinian refugee camps, towards the use of mHealth as a complement to traditional NCD care practiced in PHCCs. Factors associated with the individuals’ feedback on SMSs were also explored. This study will help understand how to improve, refine, and refocus SMS interventions for potential scale-up and to inform planning for future studies on the effectiveness of SMSs in these contexts.^37^

## Methods

### Study design

This study employs a mixed-methods design in which the effectiveness of the eSahha SMSs was evaluated quantitatively and qualitatively. The quantitative assessment was performed through phone surveys using a semi-structured questionnaire. The qualitative assessment was conducted through a series of focus group discussions (FGDs) with hypertensive and/or diabetic individuals enrolled in the eSahha intervention. The ethics review for this study was approved by the Institutional Review Board of the American University of Beirut (Protocol approval number: FHS.SS.11).

### Data collection

Upon completion of the 1-year intervention period, the 1390 individuals receiving the eSahha SMSs were contacted for a phone survey to determine their feedback on perceived usefulness and satisfaction with the service, as well as self-reported perceptions of lifestyle modifications. Phone surveys using a post-intervention semi-structured questionnaire were conducted by a research assistant between December 2016 and February 2017. To maximize participation, at least three call attempts were made. Written consent to participate in this study was originally sought from participants during the initial community-based screening phase, during which participants agreed to receive SMSs and to fill the post-test phone survey. Oral consent was taken in cases where the participants were illiterate. During phone surveys, the aim of the study, the reason for the call and the voluntary participation were reiterated.

For the quantitative phone survey, questions were set in English translated into Arabic using simplified language, then back translated to examine the validity of the translation. The questionnaire was kept short, taking ~2 min to complete; phone calls were scheduled between 9:00 am and 5:00 pm during weekdays. The phone surveys targeted directly the individuals with hypertension and/or diabetes receiving the SMSs or the relatives receiving the SMSs, whose responses were used as proxy indicators for the hypertensive and/or diabetic individuals’ views.

The first section of the questionnaire pertained to socio-demographic and economic information, as well as self-reported diseases. In the second section, the individuals’ experiences with the weekly SMSs were explored on the clarity the frequency and the perceived usefulness of SMSs received. The final question was about the perceived improvement in self-management or lifestyle modifications.

For the qualitative component of this study, five FGDs (one in each intervention center were conducted with 39 enrolled individuals. Lebanese hypertensive and/or diabetic individuals receiving the eSahha SMSs, aged 40 years or more, and residing in the catchment areas of these intervention centers were eligible to participate in the FGDs. The FGDs excluded the immediate relatives in cases where the affected individuals did not own mobile phones. Aspects not covered in the phone survey as well as themes that required further investigation as per the findings of the phone surveys were addressed in the FGDs in order to formulate a better understanding and better guide the offering of similar interventions in the future. Patients who have been part of the intervention (i.e. receiving SMSs), were invited to participate in FDGs using Arabic recruitment flyers posted at the intervention PHCCs located in rural areas. Interested participants registered their names and the researchers randomly selected eight participants per FGD. Equal representation of both genders was ensured to allow for drawing conclusions about the gender effect on access to services and the uptake of mHealth interventions. Participation was completely voluntary and a written informed consent for participation and audiotaping was obtained from all participants before FGD initiation. A semi-structured interview guide solicited the feedback of participants on the various aspects of the mhealth program. A qualified moderator facilitated the 40–60-min long FGDs in Arabic. Extensive notes were taken and discussions were audiotaped with participants’ consent to be transcribed for analysis. No identifiers were used in subsequent analyses.

### Data analysis

Descriptive statistics (frequency and percentage) were generated for survey respondents. In addition, the socio-demographic and clinical characteristics of respondents who reported receiving and reading the SMSs were compared to those reporting that SMSs were not read or not delivered to target, and differences were assessed using the chi-squared test. Views of participants (or their proxies) receiving and reading the weekly informative solely or both the weekly and reminder SMSs were then calculated. Answers to each individual SMSs’ satisfaction item were analyzed using descriptive statistics. Statements in the free-text option giving respondents the opportunity to describe their lifestyle modifications/behavior change were read thoroughly by two researchers and categorized into main themes. Bivariate analysis was conducted to test the association of SMSs’ satisfaction items (dependent variables) with participants’ characteristics (independent variables), using the chi-squared test. All statistical analyses were carried out using IBM SPSS Statistics version 24.0. Significance levels were set at a *P*-value ≤0.05.

The five FGDs were transcribed verbatim. Two researchers separately interpreted and explained the resulting transcripts through qualitative thematic analysis using Excel coding with the grounded theory approach. Resulting codes were reviewed by three team members for consistency and analyzed for categories and themes.

## Results

### Participants’ socio-demographic and clinical characteristics

A total of 1000 out of 1 390 contacted participants responded (response rate = 71.9%) to the phone survey. About 566 of the 1390 were the targeted individuals with hypertension and/or diabetes, while 434 were designated immediate relatives assigned to receive the SMSs on behalf of individuals who weren’t phone owners. In 390 cases, either the number was wrong or there was no answer.

**Fig. 1:**
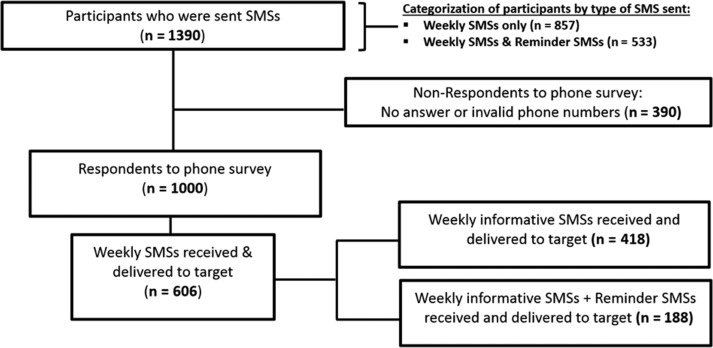
Participant flow.

Among the 1 000 respondents, 606 reported receiving and reading the SMSs. The remaining 394 reported either receiving the SMSs but not delivering them to the targeted individuals with hypertension and or/diabetes, receiving SMSs but not opening them, or not receiving the SMSs at all (Fig. 1).

The socio-demographic and clinical characteristics of respondents in the ‘SMS delivered to target’ group (*n* = 606) were similar to those of the ‘SMS not delivered to target’ group (*n* = 394), with regards to marital status, insurance status, setting (rural/refugee camp), and reason for SMSs (Table 2). Age, education status and employment status were significantly associated, in both gender groups, with receiving and reading the SMS. Younger age-group of 40–50 years were more likely to have received and read the SMS (30.3% in the received and read groups vs 14.8% in the not received/not delivered group, *P*-value < 0.001), whereas older groups (76 years or more) were more likely to have not received SMS (7.2% in the received group and 15.3 in the not received/not delivered group). This trend was more prominent among men compared to women. Higher educational levels were positively associated with receiving and reading the SMS, (25.9% high school in received vs 12.8% in not received, 9.1 university degree vs. 4.3; respectively, *P*-value < 0.001). A similar trend was observed in both gender groups (*P*-value < 0.001), with a wider difference at the lower end of education among females (among females 25% illiterate in the received group vs. 43.3% not received, among male 4.5% vs. 16.6%; respectively). A higher percentage of employed individuals were observed in those that received the SMS (32.0%) compared to those that did not (13.7%) (*P*-value < 0.001), with similar trend in both gender groups. However, men were more likely to be employed compared to women.

**Table 2:** Gender-based comparison of the demographic characteristics among those who received and read SMS and those who did not receive or did not deliver the SMS to target

	Women (*N* = 555)		Men (*N* = 444)		Total	
	SMS received & read	SMS not received/not delivered to target	SMS received & read	SMS not received/not delivered to target	SMS received & read	SMS not received/not delivered to target
	*N* (%)	*N* (%)	*N* (%)	*N* (%)	*N* (%)	*N* (%)
**Total nb of individuals**	316 (56.9)	239 (43.1)	290 (65.3)	154 (34.7)	606 (60.6)	394 (39.4)
**Age group**						
40–50	102 (32.8)	38 (15.9)	78 (27.5)	20 (13.2)	180 (30.3)	58 (14.8)
51–65	129 (41.5)	116 (48.5)	159 (56)	68 (44.7)	288 (48.4)	184 (47.1)
66–75	48 (15.4)	56 (23.4)	36 (12.7)	33 (21.7)	84 (14.1)	89 (22.8)
76 years or more	32 (10.3)	29 (12.1)	11 (3.9)	31 (20.4)	43 (7.2)	60 (15.3)
*P*-value	<0.001^a^		<0.001^a^		<0.001^a^	
**Marital status**						
Single	22 (7)	11 (4.7)	5 (1.7)	4 (2.6)	27 (4.5)	15 (3.9)
Married	204 (64.6)	151 (64.3)	264 (91)	138 (90.2)	468 (77.2)	289 (74.5)
Divorced/separated	11 (3.5)	5 (2.1)	6 (2.1)	1 (0.7)	17 (2.8)	6 (1.5)
Widowed	79 (25)	68 (28.9)	15 (5.2)	10 (6.5)	94 (15.5)	78 (20.1)
*P*-value	0.423		0.573		0.177	
**Education status**						
Illiterate	79 (25)	97 (43.3)	13 (4.5)	25 (16.6)	92 (15.2)	122 (32.5)
Reads and writes	28 (8.9)	24 (10.7)	19 (6.6)	40 (26.5)	47 (7.8)	64 (17.1)
Elementary	128 (40.5)	70 (31.3)	127 (43.8)	55 (36.4)	255 (42.1)	125 (33.3)
High School	66 (20.9)	28 (12.5)	91 (31.4)	20 (13.2)	157 (25.9)	48 (12.8)
University degree	15 (4.7)	5 (2.2)	40 (13.8)	11 (7.3)	55 (9.1)	16 (4.3)
*P*-value	<0.001^a^		<0.001^a^		<0.001^a^	
**Employment status**						
Unemployed	281 (88.9)	224 (98.2)	131 (45.2)	103 (68.2)	412 (68)	327 (86.3)
Employed	35 (11.1)	4 (1.8)	159 (54.8)	48 (31.8)	194 (32)	52 (13.7)
*P*-value	<0.001		<0.001		<0.001	
**Insurance status**						
Not insured	137 (43.4)	92 (38.5)	121 (41.7)	57 (37)	258 (42.6)	149 (37.9)
Insured (Public/Private insurance, UNRWA, Others)	179 (56.6)	147 (61.5)	169 (58.3)	97 (63)	348 (57.4)	244 (62.1)
*P*-value	0.259		0.361		0.148	
**Setting**						
Rural Area	183 (57.9)	151 (63.2)	168 (57.9)	98 (63.6)	351 (57.9)	249 (63.4)
Refugee Camp	133 (42.1)	88 (36.8)	122 (42.1)	56 (36.4)	255 (42.1)	144 (36.6)
*P*-value	0.221		0.264		0.098	
**Reasons for SMS**						
Diabetes	47 (14.9)	37 (15.5)	47 (16.2)	19 (12.3)	94 (15.5)	56 (14.2)
Hypertension	173 (54.7)	138 (57.7)	165 (56.9)	103 (66.9)	338 (55.8)	241 (61.3)
Both	96 (30.4)	64 (26.8)	78 (26.9)	32 (20.8)	174 (28.7)	96 (24.4)
*P*-value	0.649		0.123		0.208	

Some numbers under some categories may not add up to the total due to missing values.

^a^Refers to Statistical Significance at 0.05 CI.

### Survey participants’ views on eSahha SMSs

The perceptions and satisfaction with weekly and reminder SMSs were assessed among individuals who stated they received and read them (*n* = 606). Concerning the content of the eSahha SMSs, the majority agreed that the content was very easy/easy to read and understand (93.9%) and very useful/useful (93.9%) (Table 3). About 76.9% of respondents reported applying the eSahha SMSs in their daily lives (most of the times/always). Among those who received and read reminder SMSs (*n* = 188), 93.1% stated that SMSs was very useful/useful.

**Table 3:** Participants’ views on weekly (*n* = 606) and reminder SMSs (*n* = 188)

Item	N	%
***Weekly SMS (N = 606)***		
**Easiness**—SMSs are easy/very easy to understand	569	93.9
**Usefulness**—SMSs are useful/very useful	569	93.9
**Application**—SMSs are applied most of the times/always in daily life	465	76.9
***Reminder SMS (N = 188)***		
**Usefulness**—Reminder SMSs are useful/very useful	175	93.1
*Both types of SMSs*		
**Behavior change**—After receiving SMSs, there is improvement in managing the disease condition (to a great extent)	179	29.6

### Lifestyle modifications and behavior change

Through the analysis of respondents’ open-ended response statements on lifestyle modifications, 682 individual responses were received withthe following main themes emerging: SMS lead to making healthy food choices (50.6%), SMSs are effective in improving compliance to diabetes/hypertension treatment (23%), SMSs provide new information and remind us to perform healthy behaviors (7.2%) and SMSs improve adherence to medication regimens (5.3%).

### Association of SMSs’ satisfaction items with participants’ characteristics

Differences between participants in their satisfaction with weekly and reminder SMSs were assessed and presented in Table 4. For the five SMSs’ satisfaction items, there were no statistically significant differences (*P* > 0.05) when comparing participants’ sex, marital status, employment status, insurance status and reason for SMS (diagnosis). On the contrary, younger individuals aged 40–50 years reported applying SMSs more than older age groups (81.6% for 40–50 years, 78.1% for 51–65, 65.5% for 66–75 and 69.8% for 76 years or more; *P* = 0.026). Furthermore, there was a statistically significant difference in the easiness, usefulness, and application of SMSs across educational status (*P* = 0.001, *P* = 0.001 and *P* = 0.033; respectively). Additionally, participants residing in Palestinian refugee camps reported higher levels of SMSs application compared to respondents from Lebanese rural areas (82.3% and 72.9%, respectively; *P* = 0.004). Similarly, there was a significant difference in behavior change across setting, whereby 32.7% of respondents from refugee camps reported perceived lifestyle modifications/behavior change, compared to 27.4% of respondents from rural areas (*P* < 0.001).

**Table 4:** Association of SMSs’ satisfaction items with independent variables

	Items				
Variable (*N*, %)	Easiness	Usefulness	Application	Usefulness (reminder SMS)	Behavior change
**Gender**					
Male	274 (94.1)	273 (94.1)	232 (80.3)	94 (95.9)	88 (30.6)
Female	296 (93.7)	296 (93.7)	233 (73.7)	81 (90.0)	91 (28.8)
*P-value*	0.810	0.810	0.154	0.110	0.650
**Age groups**					
40–50	183 (95.8)	183 (95.8)	155 (81.6)	60 (92.3)	63 (33.0)
51–65	270 (93.8)	270 (93.8)	225 (78.1)	77 (96.3)	89 (31.1)
66–75	79 (94.0)	79 (94.0)	55 (65.5)	26 (83.9)	20 (23.8)
76 years or more	37 (86.0)	37 (86.0)	30 (69.8)	12 (100.0)	7 (16.3)
*P-value*	0.145	0.145	0.026^a^	0.100	0.247
**Marital status**					
Single	24 (88.9)	24 (88.9)	20 (74.1)	6 (100.0)	4 (14.8)
Married	442 (94.4)	442 (94.4)	364 (77.9)	136 (94.4)	147 (31.5)
Divorced/Separated	17 (100.0)	17 (100.0)	16 (94.1)	7 (100.0)	7 (41.2)
Widowed	86 (91.5)	86 (91.5)	65 (69.1)	26 (83.9)	22 (22.6)
*P-value*	0.323	0.323	0.323	0.141	0.240
**Educational status**					
Illiterate	80 (87.0)	80 (87.0)	61 (66.3)	24 (88.9)	20 (22.0)
Reads and writes	41 (87.2)	41 (87.2)	30 (65.2)	12 (80.0)	10 (21.3)
Elementary	240 (94.1)	240 (94.1)	195 (76.5)	69 (93.2)	71 (28.0)
High school	156 (99.4)	156 (99.4)	131 (83.4)	51 (98.1)	157 (36.3)
University degree	52 (94.5)	52 (94.5)	48 (87.3)	19 (95.0)	20 (38.2)
*P-value*	0.001^a^	0.001^a^	0.033^a^	0.215	0.068
**Employment status**					
Unemployed	384 (93.2)	384 (93.2)	306 (74.3)	104 (91.2)	114 (27.7)
Employed	185 (95.4)	185 (95.4)	159 (82.4)	71 (95.9)	65 (33.8)
*P-value*	0.301	0.301	0.087	0.213	0.076
**Insurance status**					
Not insured	241 (93.4)	241 (93.4)	195 (75.6)	60 (95.2)	75 (29.2)
Insured (Public/Private Insurance, UNRWA, Others)	328 (94.3)	328 (94.3)	270 (77.8)	115 (92.0)	104 (30.0)
*P-value*	0.669	0.669	0.274	0.409	0.637
**Setting**					
Rural area	326 (92.9)	326 (92.9)	256 (72.9)	95 (95.0)	96 (27.4)
Refugee camp	243 (95.3)	243 (95.3)	209 (82.3)	80 (90.9)	83 (32.7)
*P-value*	0.220	0.220	0.004^a^	0.270	<0.001^a^
**Reason for SMS**					
Diabetes	85 (90.4)	85 (90.4)	76 (81.7)	38 (97.4)	25 (26.6)
Hypertension	320 (94.7)	320 (94.7)	264 (78.1)	88 (94.6)	101 (30.1)
Both	164 (94.3)	164 (94.3)	125 (71.8)	49 (87.5)	53 (30.5)
*P-value*	0.306	0.306	0.208	0.122	0.672

^a^Refers to Statistical Significance at 0.05 CI.

### Focus group discussions with recipients of eSahha SMSs

The FGD sessions held with hypertensive and/or diabetic individuals receiving eSahha SMSs helped evaluate the participants’ experiences with the SMS service within their respective contexts in order to assess the acceptability of the intervention. Women (61.5%) were highly represented among participants of the FGDs. With regards to age, marital status, and employment, the majority of participants belonged to the age group of 40–59 years (71.8%), were married (84.6%) and unemployed (56.4%).

Altogether, ‘high rates of satisfaction with and acceptability of the received SMSs’ was recorded among FGDs participants. As such, all FGDs participants indicated their desire to continue receiving the eSahha SMSs in the future. The FGDs also showed that the majority of recipients of the intervention were satisfied with the ‘simplicity of SMS intervention’ in terms of content, ease of read and comprehension. They also reported receiving messages at appropriate times (afternoon) and frequency (once per week). Participants also expressed ‘satisfaction with being reminded’ of important health-related issues and clinical appointments. Though some participants felt that some information was repetitive and they were aware of, yet it helped them reinforce the information and improve the management of their health condition. Furthermore, a theme related to ‘disseminating beneficial SMSs’ content’ also emerged. Participants accentuated their habits of sharing the SMSs among their families and friends to spread useful information among their beloved ones. Lastly, participants perceived the eSahha SMS service as ‘useful, effective and enjoyable’ as they received guidance on food choices and on regular physician follow-up, and they learned new information. Participants’ suggestions to improve the SMS service included desire to receive more dietary and nutrition information, further information on cardiovascular diseases and first aid, and coupling voice-messaging with text-messaging to accommodate for illiterate individuals.

## Discussion

### Main findings of this study

This study evaluated the impact of the eSahha weekly and reminder SMSs employed in Lebanese rural areas and Palestinian refugee camps from the perspective of the recipients of this intervention. It explored the views and attitudes of hypertensive and/or diabetic individuals in these settings towards the use of the SMSs intervention through their perceptions of satisfaction, usefulness and implicated lifestyle modifications upon receipt of the SMS messages.

Analysis revealed that less educated individuals were more likely to not read/open the SMSs or not to deliver it to target. This might have possible explanations including people with a lower education levels did not value the health information and thus chose not to open the messages and read it, or are less likely to own a phone as it requires a certain level of technological literacy. Additionally, only 29.6% of respondents confirmed perceiving behavior change to a great extent which raises the need to identify the sustainability of this change in behavior, depending on different aspects of one’s identity, roles and responsibilities within and beyond the household. Similar findings have been reported in literature.^32^ Moreover, unemployed individuals were found more likely to not open and read the SMSs or not receive it, although this group of the population is potentially the more vulnerable, relatively sicker and in need of more awareness compared to the employed ones as they often have attained lower educational levels.

Improving diabetes and/or hypertension self-management, medication and treatment adherence were among the emerging themes in the open-ended question on behavior change. However, there remains a need for a more accurate assessment since survey respondents self-reported having behavior change, not necessarily reflecting the impact of these SMSs. More frequent behavioral and clinical assessments are thus necessary to draw better conclusions on the impact of eSahha SMSs on lifestyle modifications and behavior change.

### What is already known on this topic

The quantitative analysis of the phone surveys revealing that most recipients found weekly SMSs easy to understand, useful, and applied in their daily lives are in line with findings from similar studies in the United States, Finland, Iraq and Saudi Arabia.^13,15,17,18,22^

The significant association between educational status and SMSs’ satisfaction were concurrent with those of previous studies indicating that illiteracy and low education levels continue to be barriers in the use of SMSs, and in reporting satisfaction with this service.^32,38,39^ Having said that, it is worth pilot testing the text messages interventions with individuals of low literacy level in future research.^40^

When considering self-reported behavior change on receipt of SMSs reminders in rural areas as compared to those refugee camps, less improvement in disease management and behavior change was reported among women, those aged 51–65 and 76 or more, unemployed and illiterate individuals or those with only elementary education.

The 81.1% of participants who perceived improvement in self-management, lifestyle medications or overall improvement attributed to the eSahha SMSs shows similar figures to a study in Scotland where 82% of the recipients of SMSs felt that they had improved their diabetes self-management.^19^ This study concurs with many previous studies, with respect to respondents’ expressing improvement in their diabetes and hypertension self-management and treatment compliance, especially those related to diet and physical activity.^10,18,20,39,41^ In contrast, two other trials examined the effect of SMSs on diet and physical activity and reported no effect.^11,17^

Regarding the qualitative analysis of the FGDs, participants’ appreciation of the SMS service was obvious and dominant. Consistent with findings from other studies,^11,15–20,22^ all FGD participants enjoyed this experience, shared their satisfaction with its content, easiness, frequency, and timing, and requested to continue receiving SMSs. Yet, it is worth noting is that none of these studies targeted refugee populations, making consequently the comparison at the level of this population invalid. Participants expressed that receiving awareness SMSs was a novel way to learn new information about their conditions and improve awareness about the importance of making healthy behaviors. Despite the positive perceptions and the high reported levels of satisfaction with the eSahha SMSs, some drawbacks were also mentioned. For instance, FGD participants were concerned about illiterate people who can potentially benefit from the useful information in SMSs, yet cannot understand it. This was a potential drawback in this study as 219 respondents reported that SMSs were not received or that the participant does not open them for illiteracy purposes. It therefore emerges as a key need to address this group in future mHealth interventions by using voice messaging.^12^

### Limitations

This study has some limitations. For the quantitative component, loss to follow-up was a substantial concern as the majority of non-respondents were unreachable due to changed numbers or switched-off mobile phones. Moreover, a good proportion of respondents (*n* = 175) reported that SMSs were not delivered to target as the patient was not the mobile phone holder. This points to the necessity of conducting mid-term assessment in future interventions to make sure that the intervention is reaching its target and to emphasize the importance and the source of SMSs. This study’s qualitative component had also some limitations concerning the small sample size and inclusion criteria that excluded participants from Palestinian refugee camps. This is because the mode of follow-up and delivery among the refugee population was designed in coordination with relevant authorities in refugee camps, which advised on phone as the channel for follow-up taking into consideration cultural and security factors in the refugee settings. Yet, the views of the latter were gathered and taken into consideration through the phone surveys. Similarly, the composition of the FGDs with the majority of participants belonging to the age group of 40–59 years and being unemployed may have sub-optimally allowed exploring the point of views of employed individuals who were more likely to receive and read the SMS as per the findings, and provide potentially more rigorous insights on the content of the SMS. A final limitation is that all answers are self-reported which introduces potential social-desirability bias.

### What this study adds

To the best of our knowledge, this is the largest community based study exploring feedback on and satisfaction with SMSs among individuals with hypertension and/or diabetes in the Middle East Region. The strengths of this study lie in its target population where it addressed individuals living in hard-to-reach areas, as well as its mixed-method design whereby we supplemented the quantitative data on satisfaction with a qualitative component from the free-text answers and FGDs.

Furthermore, this study’s sample size and response rate were acceptable and allowed us to draw significant conclusions on the effectiveness of and satisfaction with the SMSs. Examining the proportion of individuals who received and actually opened and read the SMSs shows that it was more likely for participants to open and read the weekly health awareness SMSs compared to the reminder ones. This may be hypothetically related to the fact that the Lebanese health system at the PHC level was not incorporating mHealth driven reminders for appointment, which could have potentially decreased the expectation of people to receive this kind of messages. Another explanation could be that weekly awareness SMSs regularly sent sets expectations for participants to receive this type of messages on a specific fixed time every week. Reminder SMSs on the other hand may be unexpected or more likely to be missed, especially given the remarkable number of randomly received advertisement SMSs by mobile owners in Lebanon. More qualitative analysis is needed at this level to better understand the underlying factors leading to this difference.

## Conclusion

The evaluated SMS-based mHealth intervention achieved higher reach among individuals with higher educational level, and those who belong to the age range of 40–50 years. According to targeted individuals with hypertension and/or diabetes, the eSahha SMSs were found to be generally feasible, acceptable, and satisfactory among people from Lebanese rural areas and Palestinian refugee camps. SMS messages were perceived by the targeted individuals as an educational platform and the majority wished to continue receiving the service. However, it also highlighted the need for targeted attention and a diversified educational approach to older, illiterate and unemployed individuals who were relatively less able to realize the full potential of SMS-based interventions. Such approaches can include use of voice messaging and tailoring to maximize the match between the instructional content and the participants’ characteristics.^40,42^ The use of a mixed method approach revealed crucial information for the development and design of mHealth interventions for patients with chronic diseases, as it offered a snapshot about the barriers and facilitators for using SMSs which can guide future design of interventions. Users’ perceptions and feedback regarding the eSahha SMS messages serve as a basis for the evaluation of the service and the fine-tuning of such interventions before potential scale-up. This study provides deep insight for decision makers in Lebanon about promising possibilities for the design and use of tailored mHealth interventions within the PHC context among patients with chronic diseases with different experiences, needs, and opportunities.

## Supplementary Material

Supplementary Data
