# Supplementary material for: mHealth use for non-communicable diseases care in primary health: patients’ perspective from rural settings and refugee camps
Source: J Public Health (Oxf). 2018 Oct 11;40(Suppl 2):ii52–63. doi: 10.1093/pubmed/fdy172 (PMC6294037; doi:10.1093/pubmed/fdy172)
Supplement: Supplementary Data [file fdy172_rebuttal_esahha_patient_satisfaction.docx]

Date: August 13, 2018

**Manuscript Number:** JPH-18-0171

**mHealth Use for Non-communicable Diseases Care in Primary Health: Patients’ Perspective from Rural Settings and Refugee Camps**

We would like to thank the journal’s editors and respected reviewers for the valuable feedback. We are pleased to provide below a point-by-point response to the comments raised.

Kindly note that the main manuscript was amended as per the reviewers’ comments and the marked copy is sent along with this response to reviewers’ letter. The modifications referred to in the responses below were incorporated in the marked version of the manuscript using the 'track changes' function. The page numbers refer to the clean updated version.

**Reviewer 1**

| **Major Comments** | **Responses** | **Modifications** |
| --- | --- | --- |
| **1.** This study has good potential, but the most important aspect of this study from a public health perspective is the change in behaviors and not the user satisfaction.  I would strongly recommend a major revision that looks more specifically at which behaviors changed as a result of the intervention and less at the user satisfaction, which is more broadly documented in the mHealth literature. You group "behavior change" as one variable- but this needs to be broken out and presented in greater detail. Which behaviors were statistically significantly changed in the intervention vs the control group? Which were unchanged? Were there any that were worse in the intervention group? | We thank the reviewer for the suggested refocusing of this manuscript to examine behavioral changes rather than user satisfaction. We concur with the reviewer that examining behavioral changes and outcomes is pivotal and wish to clarify that these aspects of the study have been already published recently. Kindly refer to:  1: Saleh S, Farah A, Dimassi H, El Arnaout N, Constantin J, Osman M, El Morr C, Alameddine M. Using Mobile Health to Enhance Outcomes of Noncommunicable Diseases Care in Rural Settings and Refugee Camps: Randomized Controlled Trial. JMIR Mhealth Uhealth. 2018 Jul 13;6(7):e137. doi: 10.2196/mhealth.8146. PubMed PMID: 30006326.  2: Saleh S, Alameddine M, Farah A, El Arnaout N, Dimassi H, Muntaner C, El Morr C. eHealth as a facilitator of equitable access to primary healthcare: the case of caring for non-communicable diseases in rural and refugee settings in Lebanon. Int J Public Health. 2018 Jun;63(5):577-588. doi: 10.1007/s00038-018-1092-8. Epub 2018 Mar 15. PubMed PMID: 29546440.  This study offers a unique examination of user satisfaction with low-cost mhealth interventions targeting underserved populations so that the design and implementation of similar initiatives in comparable settings could be improved. | N/A |

**Reviewer 2**

| **Comment Number** | **Major Comments** | **Responses** | **Modifications** |
| --- | --- | --- | --- |
| C1 | Dear authors, thank you for the opportunity to review your important. I feel that this manuscript can be made clearer, especially with regards to reference to the broader eSahha intervention. I hope you take my comments below constructively and that you have the opportunity to consider my suggestions and of any other reviewers. | We thank the reviewer for the constructive feedback provided. We hope the edits made based on the comments rendered the manuscript clearer to the reviewers and all other readers. | N/A |
| C2 | Line 5: consider deleting nowadays | The edit indicated in the reviewer’s comment has been made. | P.4 line 3 |
| C3 | Line 8: Primary should be capital "P" | The edit indicated in the reviewer’s comment has been made. | P.4 line 5 |
| C4 | Line 17: consider deleting "Literature suggests that" and Being with "Mobile health..." | The edit indicated in the reviewer’s comment has been made. | P.4 line 11 |
| C5 | Line 17: consider bringing up definition of mHealth from line 21 to 17 when first used | The edit indicated in the reviewer’s comment has been made. | P.4 lines 11-13 |
| C6 | Line 29: add "an" between "being" and "advanced" | The edit indicated in the reviewer’s comment has been made. | P.4 line 21 |
| C7 | Line 42: consider shortening, perhaps "Frequently under-resourced PHC Centers are the only facilities..." | The edit indicated in the reviewer’s comment has been made. | P.4 line 30 |
| C8 | Line 49: This is debatable, consider revising this statement. SMS-based mHealth interventions have reported a range of outcomes, some successful but many that have made no impact (including our group's RCT on interactive SMS reminders for patients on TB treatment). | The edit indicated in the reviewer’s comment has been made. | P.5 lines 35-43 |
| C9 | Line 31: would be helpful to know why Monday afternoon. | The time was randomly selected with the hope that the messages sent will enhance education or induce behavioral change for the rest of the week. Sticking to the same time every week was important so that the SMS recipients could anticipate the messages at the same time every week. |  |
| C10 | Line 39: first time a control group has been mentioned in the full-text but the study design has not been elaborated. It would be very appropriate to have the study design outlined from the outset to lay the context for the intervention and control groups. | The study design and random allocation of centers between intervention and control groups pertaining to the initial eSahha intervention are now explained in the first introductory paragraph about the eSahha intervention as follows:  “The eSahha project is a two-pronged mHealth interventional project targeting sixteen PHCCs in six different Lebanese governorates. The centers were randomly assigned into intervention and control groups, for a total of eight sites in each of the groups. Among each of intervention and the control groups, five PHCCs were located in rural areas, and three PHCCs were located in Palestinian refugee camps. All rural and refugee settings included were considered underserved areas with limited access to health knowledge and healthcare.” | P.5 lines 44-50 |
| C11 | Line 41: authors must be very careful to describe this clearly. "Regular care" should ideally include routine foot or eye exams, etc, so the "intervention" group would be different only in the SMS reminders, not the standard practice of care. If adherence of clinicians to standard practice and patients to scheduled visits improves, that would be desirable of course. | We concur with the reviewer that the term ‘regular care’ may be confusing. The sentence has been edited as follows:  “Individuals in the control group didn’t receive SMSs and were thus receiving the usually practiced care in the control PHCCs.”  It is worth noting that the usually practiced care in the control PHCCs does not necessarily include routine foot and eye exams. This is because the overwhelming flow of patients in the PHCCs vis-à-vis the understaffing at these centers may prevent healthcare providers from practicing according to the guidelines. Providers may often skip secondary exams related to a specific disease such as foot and eye exams in the case of diabetes. | P.6 lines 68-70 |
| C12 | Line 50: This is not easy to grasp as the study design has not been clearly outlined. What are the intervention and control groups/sites? How are the different/similar? Once that is clear, then understanding differences in disease detection and referral rates, patient compliance, and quality indicators between the two groups would help the reader assess whether the right parameters are being assessed, with what degree of accuracy or standard, etc. | We agree with the reviewer that the difference in the intervention and control groups were not outlined clearly in the manuscript.  The individuals living in the catchment areas of the PHCCs categorized as intervention sites took part of the community screening service, received necessary referrals (if already diagnosed or suspected to be diabetic and/or hypertensive), and were enrolled in the SMS intervention. In parallel, individuals living in the catchment areas of the PHCCs of the control group, did not receive community screening and referral services, nor were they enrolled in the SMS intervention. |  |
| C13 | Line 22: Insufficient information provided on study design. This is a big gap, needs to be very clear what the study design is. | We concur with the reviewer that the way we stated the specific aim of this study could have been clearer.  This manuscript aims to assess the views and attitudes of hypertensive and/or diabetic individuals enrolled in the eSahha intervention (elaborately explained in the introduction section) and residing in Lebanese rural areas and Palestinian refugee camps, towards the use of mHealth as a complement to traditional NCD care practiced in PHCCs.  A mixed-methods design was adopted in this study through: 1) phone surveys using a semi-structured questionnaire to quantitatively evaluate the perception of the targeted individuals; and 2) Focus Group Discussions (FGDs) with groups of individuals who received the mHealth intervention, to qualitatively understand their perception of the intervention.  For specific details on the design of the “eSahha” project, kindly refer to the recently published manuscripts below:  1: Saleh S, Farah A, Dimassi H, El Arnaout N, Constantin J, Osman M, El Morr C, Alameddine M. Using Mobile Health to Enhance Outcomes of Noncommunicable Diseases Care in Rural Settings and Refugee Camps: Randomized Controlled Trial. JMIR Mhealth Uhealth. 2018 Jul 13;6(7):e137. doi: 10.2196/mhealth.8146. PubMed PMID: 30006326.  2: Saleh S, Alameddine M, Farah A, El Arnaout N, Dimassi H, Muntaner C, El Morr C. eHealth as a facilitator of equitable access to primary healthcare: the case of caring for non-communicable diseases in rural and refugee settings in Lebanon. Int J Public Health. 2018 Jun;63(5):577-588. doi: 10.1007/s00038-018-1092-8. Epub 2018 Mar 15. PubMed PMID: 29546440.  We have modified the text in the main manuscript in order to properly reference the above mentioned manuscripts and clarify the specific aims of this one. | P.6 lines 116-123 |
| C14 | Line 26: consider revising "ethically approved" to "ethics review was approved" | The edit indicated in the reviewer’s comment has been made. |  |
| C15 | Line 34: 1390? How were these people selected? How many were in the intervention vs control group? | As stated in the manuscript, the 1390 individuals with validated phone numbers (out of 1433 originally subscribed in the mHealth intervention) were receiving the eSahha SMSs – these constituted the full sample size of the intervention group receiving the mHealth intervention implemented prior to the conduct of this study. All individuals belonging to the intervention group (i.e. living in the catchment area of the intervention PHCCs having mobile phones and hence receiving the mHealth intervention) were eligible for inclusion in this study of this manuscript and were contacted for the phone survey. |  |
| C16 | Line 38: Eight intervention sites, so where are the control sites? | There were eight PHCC control sites, divided as follows: five in rural areas and three PHCCs in Palestinian refugee camps. | P.5 lines 44-50 |
| C17 | Line 19: the focus group methods and analysis are more detailed than the intervention study within which these seem to have been applied. The reader is missing the bigger context of the study design. | This study is conducted post-intervention to evaluate the effectiveness of the implemented intervention from the perspective of the targeted population. Please see response to point C13 for further information on study design in recently published manuscripts.  Consequently, the methods section is restricted to the explanation of the design adopted for the purpose of this manuscript rather than the design of the intervention which was published elsewhere. |  |
| C18 | Line 32: who are these 1390? Is this all patients served at the PHCC? Unlikely given the low number. So how these 1390 were selected from the universe of PHCC patients or even eSahha patients? | The 1390 individuals were receiving the eSahha SMSs – these constitute the full sample size of the intervention group receiving the “eSahha” mHealth intervention (Please see comment C15).  All individuals belonging to the intervention group (i.e. living in the catchment area of the intervention PHCCs and receiving the mHealth intervention) were eligible for inclusion in this manuscript and were contacted for the phone survey. The total of the eligible individuals was 1390. |  |
| C19 | Results: I have read the results and find them difficult to interpret in the absence of a clear understanding of the study design. There is little indication in the results of the disease detection and referral rates, patient compliance, and quality indicators between the two groups that were highlighted on Page 5 line 50 as how the overall eSahha intervention would be assessed. | We thank the reviewer for the comment. In-depth analysis of the pre- and post- intervention disease detection and referral rates, patient compliance, and other medical quality indicators and their difference between the 2 groups (intervention vs. control) are outside the scope of this paper. Such analysis was conducted in a separate manuscript dedicated to the evaluation of the effectiveness of the mHealth intervention through examining these quality indicators:  Saleh S, Farah A, Dimassi H, El Arnaout N, Constantin J, Osman M, El Morr C, Alameddine M. Using Mobile Health to Enhance Outcomes of Noncommunicable Diseases Care in Rural Settings and Refugee Camps: Randomized Controlled Trial. JMIR Mhealth Uhealth. 2018 Jul 13;6(7):e137. doi: 10.2196/mhealth.8146.  This manuscript can be accessed through this link: <https://mhealth.jmir.org/2018/7/e137/> |  |
| C20 | I may be incorrect, but the Conclusion appears derived wholly from the focus group analysis. | We agree with the reviewer – summary of findings from the quantitative survey was added to the conclusion to ensure its comprehensiveness. |  |
| C21 | It will be very important for the authors to separate the focus group analysis from what I think is a pre-existing trial that this paper is being linked to. Perhaps there is an intervention and control group that can be appropriately described, but if these are not there or not necessary for the focus group, best to leave this analysis to patients perceptions and not assess. | We concur with the reviewer that the way the manuscript reports this study as part of the larger “eSahha” initiative may be confusing. The authors did every effort in the modified version of the manuscript to clarify the focus of the manuscript and enhance readability. |  |
| C22 | Footnote: I first read this article three weeks ago and was unable to understand what I was missing. Upon reading this article multiple times today, I realized that this is a specific analysis of perceptions of eSahha users based on a telephonic survey and focus groups. While this is clearly stated in Page 6 on line 7, I am surprised that it took me multiple readings to disassociate the broader eSahha evaluation with control and intervention arms from this patient perspective survey. I think my confusion as a reviewer reflects some references to control and intervention groups in the text, as well as the over-emphasis given here to revisiting the bigger eSahha intervention (which are described in detail elsewhere) while not sufficiently highlighting this assessment of patient perception up front. | We apologize for this confusion and hope the edited version enhanced the readability of the manuscript. |  |
